# How pregnant women living with HIV and their male partners manage men's HIV self‐testing: qualitative analysis of an HIVST secondary distribution process in Kampala, Uganda

**DOI:** 10.1002/jia2.26050

**Published:** 2023-01-19

**Authors:** Norma C. Ware, Monique A. Wyatt, Emily E. Pisarski, Brenda Kamusiime, Vicent Kasiita, Grace Nalukwago, Alisaati Nalumansi, Collins Twesigye, Jade Boyer, Agnes Nakyanzi, Faith Naddunga, Andrew Mujugira, Connie L. Celum

**Affiliations:** ^1^ Department of Medicine Brigham and Women's Hospital Boston Massachusetts USA; ^2^ Department of Global Health and Social Medicine Harvard Medical School Boston Massachusetts USA; ^3^ Harvard Global Cambridge Massachusetts USA; ^4^ Infectious Diseases Institute Makerere University Kampala Uganda; ^5^ Department of Global Health University of Washington Seattle Washington USA

**Keywords:** HIV prevention, HIV self‐testing, pregnant women, secondary distribution, HIV service delivery, Uganda

## Abstract

**Introduction:**

Increased HIV testing by men in sub‐Saharan Africa is key to meeting UNAIDS 2025 testing targets. Secondary distribution of HIV self‐testing (HIVST) kits by pregnant women attending antenatal care to male partners has been shown to increase testing among African men. A detailed understanding of how women and male partners manage the distribution and use of HIVST and subsequent linkage to clinic‐based follow‐up can inform implementation and scale‐up efforts.

**Methods:**

We use qualitative data from the Obumu Study, a randomized trial of secondary distribution of HIVST by pregnant women living with HIV to male partners in Kampala, Uganda, to unpack the HIVST delivery process. The protocol included a clinic visit by male partners to confirm HIVST results. Individual interviews eliciting data on experiences of delivering and using HIVST and of subsequent linkage to clinic‐based testing were conducted with a purposefully selected sample of 45 women and 45 male partner Obumu Study participants from November 2018 to March 2021. Interview data from 59 participants (29 women and 30 men) in the HIVST arm were analysed through coding and category construction.

**Results:**

Women living with HIV were apprehensive about delivering HIVST to their partners, especially if they had not disclosed their HIV status. They invested effort in developing strategies for introducing HIVST. Male partners described a range of responses to receiving the self‐testing kit, especially fear of a positive test result. Women reported leading the self‐testing process, often conducting the test themselves. Most women confidently interpreted HIVST results. However, they tended to defer to healthcare workers rather than report positive results directly to partners. Women told their partners the testing process required a clinic follow‐up visit, often without explaining the visit's purpose. Many partners delayed the visit as a result. Women again responded by strategizing to persuade their partners to link to follow‐up care.

**Conclusions:**

Secondary distribution of HIVST by pregnant women living with HIV to male partners can be challenging, especially when women have not disclosed their HIV status. Additional support may alleviate the burden; outreach to male partners may facilitate linkage to confirmatory testing and HIV care or prevention.

## 1 INTRODUCTION

Increasing rates of HIV testing by men in sub‐Saharan Africa is key to meeting the UNAIDS 2025 target calling for 95% of individuals living with HIV to know their HIV status [1]. Routinely collected data on HIV testing in sub‐Saharan Africa consistently reveal higher testing rates for women than for men [2].

Secondary distribution of HIV self‐testing (HIVST) kits by pregnant women attending antenatal care to their male partners has emerged as a promising strategy for increasing HIV testing among African men. A considerable body of research has demonstrated the approach to be acceptable [3, 4, 5, 6, 7], feasible [8] and effective in increasing male partner self‐testing rates [9, 10, 11, 12, 13, 14, 15]. In Uganda, the distribution of HIVST by women attending antenatal care doubled testing uptake by male partners over 3 months of follow‐up, compared to the standard of care [13]. It is not yet clear whether self‐testing facilitates subsequent linkage to HIV care or prevention for men following the use of HIVST [12, 15, 16].

The available research literature also identifies barriers that can complicate women's delivery of self‐testing kits and undermine HIVST use by male partners. Prominent among these are women's fear their partners will test positive, resulting in negative consequences for the partnered relationship [17, 18, 19]. Women living with HIV who have not disclosed their status fear abandonment and associated loss of economic support stemming from unwanted disclosure in the process of delivering HIVST [20]. Additional information is needed to determine whether the secondary distribution of HIVST to male partners is feasible and appropriate for use by pregnant women living with HIV.

In 2016, WHO issued updated guidelines to support scale‐up of HIVST as part of national HIV prevention and treatment programmes [21, 22]. A number of sub‐Saharan African countries have since incorporated self‐testing into national HIV testing policies and programmes [23, 24, 25]. A detailed understanding of how users manage and follow up on self‐testing can inform implementation and scale‐up efforts. We offer a detailed view of how pregnant women living with HIV and their male partners managed delivery and use of HIVST, as well as subsequent linkage to clinic‐based follow‐up, as part of the Obumu Study in Kampala, Uganda.

## 2 METHODS

### 2.1 The Obumu Study

The Obumu Study (“strength through togetherness” in Luganda, the local language) was a randomized trial of secondary distribution of oral HIV self‐test kits (intervention), compared to letters of invitation for fast‐track HIV testing at the clinic (standard of care) for male partners of 500 pregnant women living with HIV and receiving antenatal care in Kampala, Uganda (ClinicalTrials.gov, NCT03484533). HIVST was through serial rapid testing (Determine^®^ HIV1/2, Alere Medical Company, Chiba, Japan).

The Obumu Study took place at Kitebi Health Center III, a public antenatal clinic in Kampala. All men who accompany their partners to antenatal visits are offered HIV testing as part of routine care. Women are also routinely provided with HIVST to take to their partners at home.

The Obumu Study protocol called for a clinic visit by male partners to confirm HIVST results. Men who presented at the clinic after receiving HIVST or the letter of invitation were offered oral emtricitabine‐tenofovir as HIV pre‐exposure prophylaxis (PrEP) or tenofovir‐lamuvidine‐efavirenz as antiretroviral treatment (ART). The study goals were to determine whether: (1) HIVST and the offer of PrEP increased testing rates and PrEP initiation for men (with negative test results); and (2) HIV testing combined with PrEP and ART use by male partners increased post‐partum ART adherence for women participants. Enrolment of women began in November 2018; follow‐up visits ended in September 2021. Results of the trial are reported elsewhere [26].

### 2.2 The qualitative research

This paper reports results from the analysis of qualitative data collected as part of the Obumu Study.

#### 2.2.1 Sampling and recruitment

We used a purposeful sampling of Obumu Study participants to identify 45 women and 45 male partners for the qualitative research. Our goal was to represent variation across several dimensions: disclosure of HIV status (women), length of study participation, time in the partnered relationship and HIV serostatus (men). Generally, we selected individuals rather than couples. However, in a few cases, we deliberately sampled partners of male and female participants. We oversampled participants in the intervention arm to be consistent with the 2:1 randomization scheme of the parent study. Study clinical staff facilitated contact between prospective qualitative participants and research assistants (RAs), who reached out to explain the qualitative research and invite participation.

#### 2.2.2 Qualitative data collection

Individual interviews were carried out with Obumu qualitative participants by trained Ugandan RAs. Women's interviews covered: (1) experiences of the current/recent pregnancy; (2) quality of the partnered relationship; (3) disclosure of HIV status to the male partner; (4) views on HIVST/letter of invitation as an intervention to promote male partner testing; (5) experiences of delivering HIVST/letter of invitation; (6) male partner response to HIVST/letter of invitation; and (7) partner follow‐up to HIVST/letter of invitation. Interviews with male partners covered: (1) the quality of the partnered relationship; (2) experience with HIV testing; (3) experiences of receiving HIVST/letter of invitation; (4) using the self‐testing kit (HIVST arm only); and (5) follow‐up for clinic‐based HIV testing.

Interviews were carried out in Luganda or in English, in convenient, private locations. They were scheduled at least 2 months after women were given the self‐testing kits (at Obumu Study enrolment), to ensure ample time for the delivery process to take place before the interview. Interviews lasted 60 minutes on average, and were audio‐recorded, with permission. Interviewees were reimbursed at a rate of 30,000 Ugandan shillings, consistent with local IRB practices. Audio recordings were transcribed directly into English by the RA who conducted the interview. Interview transcripts were regularly reviewed to monitor data quality. RAs participated in weekly calls and emails for feedback on interviewing and transcription technique. Qualitative data collection began in November 2018 and ended in March 2021.

#### 2.2.3 Analysis of qualitative data

To reduce the data, we developed a coding scheme and coded the entire data set. First, we used open coding to identify and label relevant content. Labels were operationally defined, reviewed, refined and assembled into a codebook. To ensure coding consistency, two coders each coded a subset of the transcripts (20%), then identified and resolved discrepancies through discussion. The entire data set was then coded using Dedoose qualitative data management software (https://www.dedoose.com/).

The analysis reported here draws upon interview data from male and female qualitative study participants in the HIVST trial arm. The goal was to construct a detailed account of HIVST delivery and use and of subsequent linkage to clinic‐based confirmatory testing by male partners.

For this inductive analysis, author NCW reviewed coded data focusing on participants’ accounts of their perceptions of and experiences with HIVST, and examined selected complete transcripts to contextualize coded material. The review yielded a series of concepts representing major steps in the HIVST delivery process. The concepts served as the basis for developing a set of corresponding categories using the coded data. The conceptual categories provide a detailed description of the process of HIVST delivery, use and subsequent linkage to clinic‐based HIV testing as these unfolded among qualitative research participants in the Obumu Study.

### 2.3 Ethical approval

Approval to carry out the qualitative research was obtained from the University of Washington Institutional Review Board, Seattle, USA; the National HIV/AIDS Research Committee, Kampala, Uganda; and the Uganda National Council for Science and Technology, Kampala, Uganda. Participants provided consent for qualitative interviews as part of the Obumu Study consent process. Consent was re‐confirmed verbally as part of recruitment for the qualitative interviews.

## 3 RESULTS

The analysis reported here is based on in‐depth individual interviews with the 29 women and 30 male partner qualitative participants in the HIVST arm.

### 3.1 Characteristics of the qualitative HIVST arm sample

The median age of women qualitative participants in the HIVST arm was 27 years at Obumu Study enrolment; the median age of male partners was 32 years. The median length of the partnered relationship was 3 years for women and 3.5 years for men. Eighty percent of men were members of serodifferent couples. For women, the median length of time since HIV diagnosis was 1.75 years. The median time passed since Obumu Study enrolment (and women's receipt of HIVST) at the qualitative interview was 3 months for women participants and 6 months for male partners.

A third (34%) of women participants reported having disclosed their HIV‐positive status to their partners at enrolment in the randomized trial; slightly more than half (52%) of women qualitative participants reported having disclosed their HIV status to their partners by the qualitative interview. Nineteen (63%) of 30 male partner qualitative participants in the HIVST intervention arm reported knowing their partner was living with HIV.

Twenty‐five (86%) women in the HIVST arm qualitative sample reported having delivered the self‐testing kit to their partners at the time of the qualitative interview. Fourteen (48%) of these 25 women reported the kits they delivered had been used. Twenty‐five (83%) of the 30 men in the qualitative sample reported having received the self‐testing kit from their partners. Of these, 20 (80%) reported they had used the self‐testing kit (Table 1).

**Table 1:** Characteristics of the HIVST arm qualitative sample

	Women participants (*N* = 29) Median (IQR) or *N* (%)	Male partner participants (*N* = 30) Median (IQR) or *N* (%)
Age (years)	27 (25–30)	32 (29–35)
Length of relationship (years)	3 (2–7)	3.5 (1.75–6.75)
Member of serodifferent couple	N/A	24 (80%)
Time since first HIV diagnosis (years)	1.75 (0.05–5)^a^	N/A
Time since enrolment at qualitative interview (months)	3 (2–12)	6 (2–18)
Report disclosure of HIV‐positive status at Obumu Study enrolment	10 (34%)	N/A
Report disclosure of HIV‐positive status by qualitative interview	15 (52%)	19 (63%)
Report HIVST delivered to male partner at qualitative interview	25 (86%)	25 (83%)
Report HIVST kit was used	14 (48%)	20 (of 25) (80%)

^a^ Data on time since first HIV diagnosis are missing for one participant.

### 3.2 Qualitative results

Following are five conceptual categories laying out the results of our detailed analysis of the process of delivering and using HIVST in the Obumu Study, and of men's subsequent linkage to clinic‐based confirmatory HIV testing:

1. *Women's First Thoughts on Receiving HIVST and Preparation for Delivery of Self‐Testing Kits*. Women's initial reactions varied when they were presented with the self‐testing kit by Obumu Study staff. Some welcomed the opportunity to learn their partner's current HIV status. Many women, however, found the prospect of delivering the kit daunting, and viewed the task of introducing HIVST to their partners as requiring careful thought and planning. Some spent weeks working up the courage to deliver the self‐testing kit. Women who had not disclosed their HIV status in particular expressed apprehension, fearing HIVST would lead to unwanted disclosure and subsequent abandonment by their partners, upon whom they depended for financial support.
  “I was scared [to deliver HIVST] because I knew that if he gets to know my status he will ask me to leave his home and that will be the start of my suffering. Now that we are in a discordant relationship, I am more scared that he cannot be patient with me. This situation of discordance makes it so hard for me to talk to him about my status. When I tell him that I am positive when he is negative, he will say, ‘Go to your parents and leave me alone’. I will suffer, I will even get stressed and end up dying.” Woman, Age 25
2. *Introducing HIVST to Male Partners*. Women received information about HIVST from Obumu Study staff, including suggested techniques for introducing HIVST to their partners. This counselling is likely reflected in women's frequent reports of using “strategies” for presenting the kits. One strategy was to point out the advantages of self‐testing—the opportunity to save time by avoiding a clinic visit, and to test without a finger prick, for example. Some women acknowledged they had invoked clinic staff as an “authoritative voice,” arguing their partners should use HIVST because “the health workers want them to.” A few explained how they had deliberately worked to create a favourable atmosphere before introducing the self‐testing kit, as in the following:
  “I had to handle him with a lot of care before I could give him the kit to use. …I gave him everything he wanted in time. Supper was served in time and before that we had good stories and things like that…” Woman, Age 31

Women participants varied in how they handled the task of informing their partners the kit was a test for HIV. Those who enjoyed easy communication in the partnered relationship, and/or who had disclosed their own HIV status, could be forthright in acknowledging the test's purpose. Others, however, feared delivering the kit would prompt questions about their own testing. These women felt constrained to look for delivery strategies that would allow them to avoid discussing HIV, such as indicating the test had another purpose (e.g. screening for syphilis), or leaving the kit, without explanation, in a place where they hoped their partner would find it. If she could see no alternative, a woman might acknowledge she had been tested for HIV as she presented the kit to her partner, but choose to misrepresent her HIV status, as in the example below:

“I told him that this kit tests HIV so you need to test for them to know your status since I was tested and I am negative.” Woman, Age 35

3. *Male Partner Responses to Receiving an HIVST Kit from Women Participants*. Male partners described a range of reactions to receiving an HIVST kit from women participants. At the extremes were “enthusiasm” and “resistance.” A few men declared themselves “delighted” to have the chance to test at home; a few others explained that they ignored the self‐testing kit after receiving it, became angry with their partners and/or put off using the test. Between these two extremes lay a number of intermediate responses. Prominent among these was fear that the result of the test would be positive.

When asked to explain this fear, men cited feeling at increased risk due to past unprotected sex. They also worried about being ostracized from the community upon being identified as living with HIV. The overriding reason for men's fear, however, was the meaning they attached to HIV disease. Even though most Ugandans are now aware that treatment for HIV is available and effective, men in this sample associated HIV infection with early death. They said:

“The first thing that came to mind [when I tested positive for HIV] is ‘I'm going to die. I may not last a week’. As long as these thoughts surface in one's head, they get fear. The main cause of not testing is fear.” Male Partner, Age 40

“When they tell you [that] you have HIV the next thing you think about is death. It is so scaring and worrying when one gets to know one has HIV. That is why men don't want to test.” Male Partner, Age 32

4. *Using HIVST*. Women and male partner interviewees who reported using HIVST provided detailed descriptions of these experiences. Their accounts revealed a tendency for women to lead the self‐testing process, since they were more familiar with the self‐testing kit, having been instructed in its use by Obumu Study staff. Typically, women reported explaining to their partners how the testing worked, and then either conducting the test themselves or talking their partners through it. Rarely did men test themselves.

“I told him that the health worker had given me that thing (testing kit). I said, ‘we should test and see and when you have some time you should go there’. He replied, ‘there is no problem’. We tested. …It was me who was nearly doing everything…. Because the health worker had given me directions.” Woman, Age 25

Most women had confidence in their ability to interpret HIVST results and readily did so as part of the HIVST testing process. Some immediately communicated the results of the testing to their partners, particularly when the results were negative. Women did not always feel comfortable communicating HIV‐positive results to partners. Rather they deferred to health workers, directing their partners to visit the clinic to learn their self‐testing results.

“He asked me what the results were after the testing. I asked him what he thought the results would be and he told me that it is me they taught, so he expects me to tell him the results. I told him that if he wanted to know the results he should go to hospital and the health workers tell him the results.” Woman, Age 34

Male partners offered a number of observations on their experiences of using HIVST. There was general agreement that self‐testing was “quick, easy and painless.” Some felt that self‐testing made seeking clinic‐based follow‐up easier because they already had a sense of what their HIV status was. Increased confidence in ART as HIV prevention for men who received a negative self‐test result sometimes followed from HIVST use.

5. *Linkage to Clinic‐based Confirmatory Testing*. The Obumu Study sought to determine whether the secondary distribution of HIVST facilitated male partners’ subsequent linkage to clinic‐based confirmatory HIV testing and treatment or prevention services. As part of delivering the self‐test kits, many women told their partners the testing process required a clinic follow‐up visit, without making it clear what the purpose of the visit was. As a result, men were initially hesitant, and delayed.

In response, women again drew on strategies to try to persuade their partners to attend the clinic. One frequently used tactic involved invoking the authority of the healthcare system. For example, rather than pointing out the personal health benefits of HIV testing, women emphasized the importance of follow‐up as “compliance” with the preferences of health workers. Some women emphasized the cash reimbursement partners would receive from the Obumu Study at the follow‐up visit. Often, women sought to leverage men's responsibilities to the current pregnancy to convince them to visit the clinic:

“I told him you are needed at [name of clinic], they want to check this baby in the womb when you are also around**.”** Woman, Age 33

“I told him that every pregnant woman would have to go with their partner [to the clinic] or else they will not work on me.” Woman, Age 24

Many of the men eventually visited the clinic for confirmatory HIV testing after being asked by their partners to do so. However, not all of them understood the visit would include a test for HIV. Some men simply responded to hearing from their partners that they “were needed” at the hospital. Others acceded to their partner's urging in order to avoid conflict and keep peace at home. Many were acting on what they understood to be their responsibilities as future fathers to support their partners during pregnancy by accompanying them to antenatal visits, as indicated in the following excerpts:

“I came to the hospital because of my wife who was pregnant. You have to come with her, to escort her to hospital.” Male Partner, Age 28

“My wife was pregnant and I knew that once women are pregnant [they] are told to take their husbands.” Male Partner, Age 32

A number of male partners in our sample made the link to clinic‐based testing after receiving a phone call from a male outreach worker representing the Obumu Study. Phone outreach was carried out with permission from women participants to boost male partner participation. Male interviewees receiving an outreach call reported being asked to come to the clinic “to talk,” or to discuss issues related to their newborn child. A few said they were told it was important for them to know their HIV status as partners of pregnant women. Because the request came from a healthcare worker, specifically a male healthcare worker, these men were ultimately persuaded:

“It was a man speaking to me. I got strength to go since it was a man talking to me.” Male Partner, Age 32

## 4 DISCUSSION

**Figure 1:**
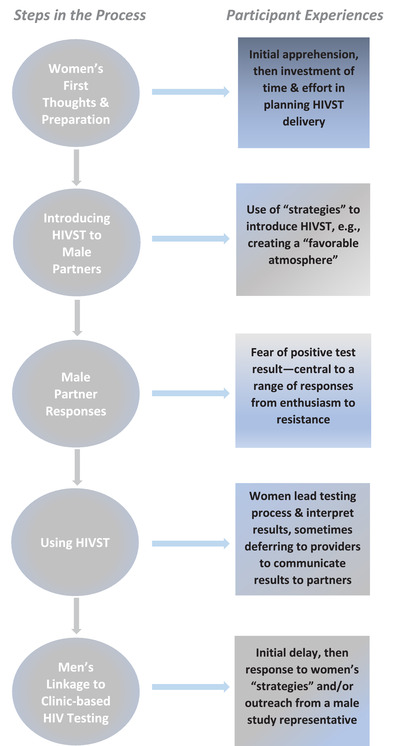
The process of delivering HIVST by Ugandan pregnant women living with HIV to male partners in Kampala, Uganda.

Many women were apprehensive at the prospect of delivering HIVST to their male partners, and invested time and effort in meeting what they experienced as a challenge. The women displayed skill and ingenuity in managing the delivery process, developing deliberate strategies to succeed in introducing the kits to their partners—including strategies for circumventing disclosure of their own HIV‐positive status. Typically, women led the self‐testing process, but not all communicated the results of testing to their partners, even though they largely understood them. Male partners reported a range of responses to receiving the self‐testing kits but were often slow to make the link to clinic‐based testing following HIVST use (Figure 1).

Prior studies of outcomes of secondary distribution of HIVST by pregnant women to male partners have demonstrated that the intervention increases partner testing. These studies have largely not focused on pregnant women living with HIV [9, 10, 11, 12, 13, 14, 15]. Much less is known about what the process of delivering HIVST, receiving HIVST and using the self‐test kit is like for these women and men. Our analysis clearly reveals the intentionality and effort women felt was required to manage the delivery and testing processes so as to avoid angry responses from their partners and negative consequences for the partnered relationship. With support from Obumu Study staff, they identified deliberate strategies for presenting self‐testing kits, and interpreting and communicating test results. These strategies were deployed with success and described in detail in interviews. This “strategic” approach to HIVST delivery and partner testing by Ugandan pregnant women is also referenced in another qualitative report [17].

Disclosure of HIV status emerged as a major barrier to HIVST delivery in this sample of Ugandan pregnant women living with HIV. Women who had not disclosed their HIV‐positive status to their partners when they undertook to deliver the self‐testing kit feared the process would trigger unwelcome questions about their HIV testing experiences, culminating in unwanted disclosure. They worried that disclosure would result in abandonment by their partners, upon whom they depended on for financial support, particularly during pregnancy. Because of these worries, women looked for ways of delivering the kit that would allow them to avoid discussing HIV, rather than using the delivery process as an opportunity to disclose. In our sample, lack of disclosure tended to delay, but not prevent, women participants from delivering self‐test kits. Ugandan women's reluctance to disclose their HIV‐positive status to male partners for fear of abandonment has been reported in previous qualitative and mixed‐method studies [20, 27, 28]. Notably, some data suggest that abandonment by male partners does not necessarily result from women's disclosure of their HIV‐positive status, particularly when disclosure takes place as part of couples‐based counselling and testing [29].

The significance of fear of discovering one is living with HIV may be underappreciated as a barrier to HIV testing for men. Men in this sample clearly pointed to fear of a positive HIV test result as a driver of their reluctance to test in general and their active efforts to avoid self‐testing in particular. Completing the self‐test did not always dissipate these fears. Having the opportunity to speak informally with a male Obumu Study outreach worker appeared to encourage some men to attend the clinic and test. Others were motivated by a sense of obligation to support their spouses during pregnancy by accompanying them to antenatal visits. The influence of perceived role responsibilities on men's decisions to engage in care has been explored in depth in a separate analysis [30]. Previous research has identified fear of a positive result as an important barrier to HIV testing for Ugandan couples [31].

Secondary distribution of HIVST by pregnant women to male partners increases testing for men and is considered to be acceptable to women asked to deliver the self‐testing kits [3, 4, 5, 6, 7]. While not challenging this claim, this analysis makes clear the effort women participants in our qualitative sample invested in presenting the self‐testing kit to their partners and convincing them to use the test. How much of this effort stemmed from a felt need to avoid disclosure of their own HIV status in the process of delivering HIVST remains an open question. Our qualitative data suggest strongly that despite being acceptable, the prospect of delivering HIVST to male partners places a burden on pregnant women living with HIV.

Several qualitative investigations of the secondary distribution of HIVST by pregnant women have been described in the research literature. A cluster of these are “hypothetical” assessments of acceptability that asked women and their partners to imagine delivering, receiving and using HIVST kits and to gauge their anticipated reactions [5, 7, 32]. An early qualitative study (data collected in 2017, published in 2021) of pregnant/postpartum women's, partners’ and healthcare providers’ knowledge relevant to self‐testing has been reported for Uganda [33]. Most closely related to the present study is the analysis offered by Matovu et al. [17], which examines Ugandan pregnant women's and partners’ experiences of HIVST delivery and use. This account also highlights women's reliance on deliberate strategies to facilitate delivery and enhance men's receptivity to HIVST use. The current study enriches this existing body of work by: (1) focusing on pregnant women who are living with HIV; (2) offering a highly detailed representation of the HIVST delivery process; and (3) including data on linkage to confirmatory clinic‐based testing by male partners following the receipt of HIVST.

The burden HIVST delivery appears to represent for pregnant women living with HIV might be alleviated through the provision of additional support for the delivery process in general, and disclosure of HIV status in particular. Women participants in the Obumu Study received counselling from study staff on the nature and use of HIVST. Discussions of approaches to delivering self‐testing kits and disclosing HIV‐positive status were part of these conversations. These counselling sessions might be amplified and formalized to facilitate the delivery process. “Accompaniment,” a multi‐faceted approach to patient support that can range from providing encouragement to “walking with” someone seeking care for HIV (or another health problem), may be helpful in thinking about how to strengthen support for women who are living with HIV and delivering self‐testing kits to their male partners [34, 35].

Male partners’ apparent responsiveness to phone calls from a male Obumu Study representative suggests that outreach by clinic staff or peers may encourage men to link to facility‐based HIV testing and prevention or treatment services. Previous research in Uganda documents the success of trained lay counsellors who are members of local communities in facilitating the linkage to clinic‐based care following home‐based HIV testing [36, 37, 38]. A recently reported study of “peer‐assisted” HIVST for members of key populations (female sex workers, men who have sex with men, transgender persons) in Burundi showed that almost all (96%) participants with a reactive self‐test subsequently linked to a care facility for confirmatory testing [39].

A national lockdown instituted in Uganda in response to the COVID‐19 pandemic impeded access to the clinic for Obumu Study participants seeking HIV testing, medication refills, and antenatal and post‐natal care, and support for childbirth. In qualitative interviews, participants emphasized their success in reaching the clinic despite lockdown restrictions. Men reported walking or cycling to the clinic for testing and PrEP refills. Pregnant women and women with infants had permission to access public transportation to travel to healthcare appointments but often found it preferable to travel to the clinic on foot.

In this qualitative analysis of HIVST delivery, we have focused on the experiences of HIVST users rather than the activities of intervention implementers. Such a shift in focus allows a detailed view of implementation activities. We see in detail how pregnant women living with HIV introduce HIVST to their partners, the partners’ response, the testing process and subsequent efforts to link men to clinic‐based confirmatory testing. The focus on how users implement the intervention also reveals how it affects them. We believe this kind of detailed process analysis has an ongoing role to play in intervention development research.

This study has the following limitations. Our qualitative sample is purposefully defined, which promotes the examination of variation but means the results reported are not representative of Obumu Study participants as a whole. The Obumu Study was based at a single antenatal clinic in Kampala, Uganda, preventing us from examining the experiences of women and men in different clinics and making comparisons across services and/or locations. Finally, the possible impact of social desirability should be recognized. We use the term social desirability to refer to the tendency of research participants to shape their responses to interview questions to conform to what they believe to be the preferences of the research staff. Male partner participants in this sample, for example, might have characterized their responses to receiving a self‐test kit as more positive than they actually were. To offset this, we prioritized the elicitation of actual experiences over attitudes and opinions in qualitative interviews.

## 5 CONCLUSIONS

This qualitative analysis provides a detailed view of how Ugandan pregnant women with HIV and their male partners managed men's self‐testing in the Obumu Study. Results suggest the process may be less simple and straightforward than it appears, especially for women living with HIV who have not disclosed their HIV status. Women in this sample who had not disclosed to their partners struggled to find ways to avoid disclosure while delivering HIVST. The women overall felt constrained to invest time and energy in developing a “strategic approach” to HIVST delivery that would predispose male partners to accepting and using the kits.

Secondary distribution of HIVST by pregnant women to male partners increases testing rates among men but places a burden on women charged with delivering the self‐testing kits. Additional support for these women in the form of counselling, peer consultation and/or “accompaniment” for disclosure and delivery may alleviate this burden and enhance women's comfort and enthusiasm for partner self‐testing for HIV. Outreach strategies merit further investigation as ways of promoting men's linkage to clinic‐based care following HIV testing in the community.

## COMPETING INTERESTS

The authors declare they have no competing interests.

## AUTHORS’ CONTRIBUTIONS

NCW and MAW designed the qualitative research. MAW designed the data collection instruments. BK, VK, GNK, AN and CT collected the qualitative data. EEP, MAW and AM supervised the data collection process. EEP and JB coded the qualitative data. NCW analysed the qualitative data. MAW, EEP, CLC and AM provided early feedback on the qualitative results. NCW drafted the manuscript and made revisions. All authors read and approved the final version.

## FUNDING

This work was supported, in whole or in part, by a grant to CLC from the US National Institutes of Health [NIMH R01 MH113434].

## Data Availability

Qualitative data underlying the analysis presented here are available from the corresponding author upon reasonable request.
